# Prediction of venous thromboembolism in newly diagnosed patients treated for lymphoid malignancies: validation of the Khorana Risk Score

**DOI:** 10.1007/s12032-017-1065-4

**Published:** 2017-12-04

**Authors:** Joanna Rupa-Matysek, Lidia Gil, Maciej Kaźmierczak, Marta Barańska, Mieczysław Komarnicki

**Affiliations:** Department of Hematology and Bone Marrow Transplantation, Poznan University of Medical Sciences, Szamarzewskiego 84, 60-569 Poznan, Poland

**Keywords:** Diffuse large B cell lymphoma, Hodgkin lymphoma, Venous thromboembolism, Khorana Risk Score

## Abstract

The utility of the venous thromboembolism (VTE) risk assessment model known as the Khorana Risk Score (KRS) in patients with lymphoid malignancies receiving outpatient chemotherapy is not defined. We evaluated the association of the KRS with VTE in patients treated for diffuse large B cell lymphoma (DLBCL) or Hodgkin lymphoma (HL). Retrospective analyses were performed in 428 patients, 241 of whom were newly diagnosed with DLBCL and 187 of whom had HL. During the initial therapy, 64 (15%) patients developed VTE and 56 died during follow-up. More VTE events occurred in patients with DLBCL than in patients with HL. According to the KRS, 364 (85%) and 64 (15%) patients were considered to be at intermediate risk and high risk of VTE development, respectively. The high-risk KRS patients were more often diagnosed with HL than DLBCL (19 vs. 10%, *P* = 0.0143). The KRS did not discriminate between high- and intermediate-risk patients with respect to VTE occurrence (17 vs. 15%, *P* = 0.5868). In our patients, the KRS did not adequately predict VTE (positive predictive value 15%, negative predictive value 82% and *C* statistic 0.51). In the multivariate analysis, bulky disease (OR 2.34; 95% CI 1.62–3.36, *P* < 0.0001), poor prognostic disease (OR 1.32; 95% CI 1.01–1.74, *P* = 0.049) and DLBCL histological subtype (OR 1.61; 95% CI 1.17–2.19, *P* = 0.003) were all significantly associated with the VTE development. In this cohort of patients with lymphoid malignancies, the KRS did not adequately stratify or predict VTE events in patients at a higher risk of VTE. This finding suggests the need for the development of a disease-specific VTE assessment model.

## Introduction

Venous thromboembolism (VTE), which comprises deep vein thrombosis (DVT) and pulmonary embolism (PE), is a frequent complication in patients with cancer, and it is associated with increased morbidity and mortality [1, 2]. Based on selected biomarkers and clinical factors, some VTE assessment models for chemotherapy-associated thrombosis were proposed [3–8]. To date, the model developed by Khorana, known as the Khorana Risk Score (KRS), is the best validated model with which to stratify VTE risk in outpatients with cancer [5, 6].

The risk of thrombotic complications in patients with lymphoma is believed to be similar to that in patients with solid tumors. Furthermore, depending on other coexisting factors, this risk is estimated as intermediate or high in the Khorana VTE risk assessment model. Lymphomas represent one of the most heterogeneous groups of malignancies [9]. Due to this heterogeneity, unique biological and clinical features and different risks of thrombosis, a single-risk model may not be suitable for all subtypes of lymphomas [10]. Therefore, the question arises of whether the KRS is valid for this group of patients.

Thus, the aim of the present study was to determine the association of the KRS with VTE and all-cause mortality in patients treated for newly diagnosed diffuse large B cell lymphoma (DLBCL) and Hodgkin lymphoma (HL).

## Patients and methods

We retrospectively analyzed newly diagnosed patients with HD or DLBCL who were receiving their first-line chemotherapy for the occurrence of VTE. All studied patients were in good general condition [Eastern Cooperative Oncology Group (ECOG)/World Health Organization (WHO) performance status 0–2] and qualified for ABVD for HD and CHOP-R for DLBCL in the outpatient clinic of the Department of Hematology and Bone Marrow Transplantation at Poznan University of Medical Sciences between June 2009 and July 2016. The observation time was defined by the study end date (December 2016), disease progression and occurrence of VTE or death.

Patients who received thromboprophylaxis at the start of chemotherapy were excluded from the study. Erythropoiesis-stimulating agents were not administered. No patients underwent central venous catheter implantation during their first-line therapy.

Routine screening for VTE was not conducted. Color and Doppler ultrasonography was used to diagnose deep vein thrombosis (DVT) only in symptomatic patients, and computed tomography angiography (CTA) was performed to detect pulmonary embolism (PE).

Demographic data and clinical details (stage of disease according to the Lugano classification; the presence of constitutional symptoms; mediastinal bulky disease, defined as the longest measurement of a tumor mass of 10 cm or greater; International Prognostic Index (IPI) score for DLBCL; International Prognostic Score (IPS) for HD; and KRS) were all analyzed [11–14].

The patients were categorized into intermediate-risk (1–2 points) and high-risk (≥ 3 points) groups using the VTE risk assessment model developed by Khorana, based on the cancer site (lymphoma was categorized as high risk), pre-chemotherapy platelet count > 350 × 10^9^/l, leukocyte count > 11 × 10^9^/l, hemoglobin < 10 g/dl and/or the use of erythropoiesis-stimulating agents, and a body mass index > 35 kg/m^2^ (1 point each) [5]. For the Khorana model, a full blood count was performed by standard methods.

Because our study involved retrospective analysis of existing data with no patient intervention or interaction, and the patient data were de-identified, the Bioethics Committee of Poznan University of Medical Sciences determined that this study was not a medical experiment and was exempt from the Bioethics Committee of Poznan University of Medical Sciences review (No KB-1029/17).

### Statistical analysis

Descriptive statistics, such as the frequency (*n*), arithmetic mean ($\bar{x}$) and standard deviation (SD), are presented for normally distributed variables. Otherwise, medians and the standard errors (SE) with interquartile ranges (25 and 75 percentiles) were used. The Shapiro–Wilk test was performed to assess normality. To compare differences between the groups, the Chi-square test was used for categorical variables and the Mann–Whitney *U* test was used for continuous variables.

Univariate logistic regression was used to evaluate potential risk factors that may influence VTE. A multivariate analysis was performed with selected variables that were significant in the univariate analysis (*P* < 0.01). In each model, the odds ratio (OR) for each independent variable was determined with a confidence interval (CI) of 95%.

The probabilities of survival were estimated via the Kaplan–Meier method, and univariate comparisons were made via the log-rank test. The Cox proportional hazards model was fitted to estimate the effect of the analyzed factors on the outcome. In this model, the hazard ratio (HR) for each independent variable was determined with a 95% CI. A *P* value < 0.05 was regarded as statistically significant. The statistical analyses were performed with STATISTICA 10 and STATISTICA Medical Package 2.0 (StatSoft, Inc., Tulsa, Oklahoma, USA).

## Results

A total of 428 adult patients with newly diagnosed DLBCL (*n* = 241) or HL (*n* = 187) were enrolled in the study. All patients were Caucasian, with a median age of 50 years (range 18–98 years), and 51% were female. The median observation time was 37 months (range 0.5–92).

The majority of patients presented with advanced lymphoma (*n* = 297; 69%), and 178 (42%) patients were classified as having a poor prognosis. Patients with DLBCL were older and more often diagnosed with stage IV of the disease than the HL group (*P* < 0.001). The HL group had a greater incidence of mediastinal bulky disease than the DLBCL group (*P* = 0.008). There were no significant differences in gender distribution, the presence of constitutional symptoms or poor prognostic disease between the HL and DLBCL patients (Table 1).

**Table 1:** Patient characteristics

	Overall population *n* = 428	DLBCL^a^ *n* = 241	HL^a^ *n* = 187	*P* value
Median age, range years	50 (18–98)	60 (18–98)	36 (18–84)	< 0.0001
Gender, male *n* (%)	209	123 (51%)	86 (46%)	0.3000
Advanced disease^b^	218	158 (66%)	60 (32%)	< 0.0001
Constitutional symptoms	258	146 (61%)	112 (60%)	0.8853
Bulky disease	45	17 (7%)	28 (15%)	0.0081
Poor prognostic disease^c^	178	105 (44%)	73 (39%)	0.3455
High KRS^d^	64	25 (10%)	39 (21%)	0.0026
Presence of VTE	64	45 (19%)	19 (10%)	0.0143
Death	56	41 (17%)	15 (8%)	0.0062

*P* < 0.05: statistically significant

^a^The percentages are related to the numbers presented in the first column of the same line

^b^Advanced disease: stage according to Lugano IV

^c^International Prognostic Index ≥ 3; International Prognostic Score ≥ 3

^d^According to the Khorana Risk Score (KRS) for VTE risk assessment

In the entire study group, 64 (15%) patients developed VTE in the median follow-up period of 4.7 months (25th–75th percentile: 1.4–7.6), including 18 (28%) cases of deep vein thrombosis of the lower extremities, 7 (11%) symptomatic pulmonary embolisms and 39 (61%) cases of deep vein thrombosis at other sites (internal jugular vein—23, portal vein—1, upper extremity—15). More VTE events were found in patients with DLBCL than HL (19 vs. 10%, *P* = 0.0143), as well as in patients with rather than without bulky disease (26 vs. 8%, *P* < 0.0001; Table 2). Most of the thrombotic events occurred within 6 months after diagnosis (55%).

**Table 2:** Comparison of the characteristics of patients with and without VTE

	Overall population *n* = 428	VTE group during follow-up^a^ *n* = 64	Non-VTE group during follow-up^a^ *n* = 364	*P* value
Median age, range years	50 (18–98)	49 (22–81)	50 (18–98)	0.9698
Gender, male *n* (%)	209 (49%)	34 (53%)	175 (48%)	0.4562
Type of lymphoma: DLBCL	241 (56%)	45 (70%)	196 (54%)	0.0143
Advanced disease^b^	218 (51%)	38 (59%)	180 (49%)	0.1430
Constitutional symptoms	258 (60%)	23 (36%)	41 (64%)	0.5025
Bulky disease	45 (11%)	17 (26%)	28 (8%)	< 0.0001
Poor prognostic disease^c^	178 (42%)	34 (53%)	144 (40%)	0.0423
High KRS^d^	64 (15%)	11 (17%)	53 (15%)	0.5868
Death	56 (13%)	17 (27%)	39 (11%)	0.0005

*P* < 0.05: statistically significant

^a^The percentages are related to the numbers presented in the first column of the same line

^b^Advanced disease: stage according to Lugano IV

^c^IPI, International Prognostic Index ≥ 3; IPS, International Prognostic Score ≥ 3

^d^According to the Khorana Risk Score (KRS) for VTE risk assessment

According to the KRS, 364 (85%) patients were considered to be at intermediate risk and 64 (15%) patients were considered to be at high risk of thrombosis development. The high-risk KRS patients were more often diagnosed with HL than DLBCL (39 vs. 25, *P* = 0.0026), and they more often had constitutional symptoms (49 vs. 15, *P* = 0.0039), poor prognostic disease (41 vs. 15, *P* < 0.0001) and advanced-stage lymphoma (34 vs. 30, *P* = 0.7039). VTE occurred in 17% (*n* = 11) of the high-risk patients and in 15% (*n* = 53) of the intermediate-risk patients according to the KRS (*P* = 0.5868).

**Fig. 1:**
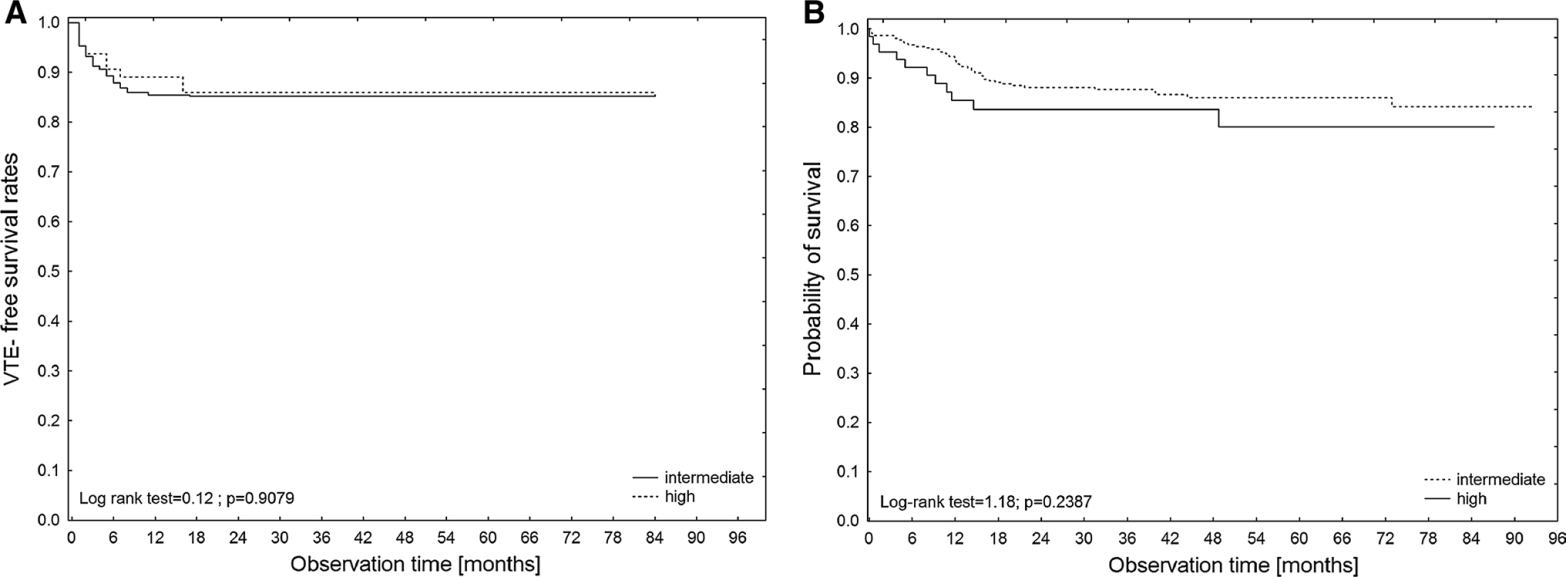
Kaplan–Meier analysis of VTE-free survival rates (**A**) and overall survival rates (**B**) according to the KRS category (high versus intermediate)

The overall cumulative incidences of VTE in patients with high and intermediate KRSs were 16.6% (95% CI 9.0–30.8) and 16.0% (95% CI 12.2–20.8), respectively (*P* = 0.9151). The cumulative incidences of VTE at 3, 6, 12 and 24 months were 8.9, 12.7, 15.4 and 16.6% in patients with a high KRS and 8.6, 12.2, 15.4 and 16.0% in patients with an intermediate KRS, respectively. These differences were not statistically significant (Fig. 1a).

**Fig. 2:**
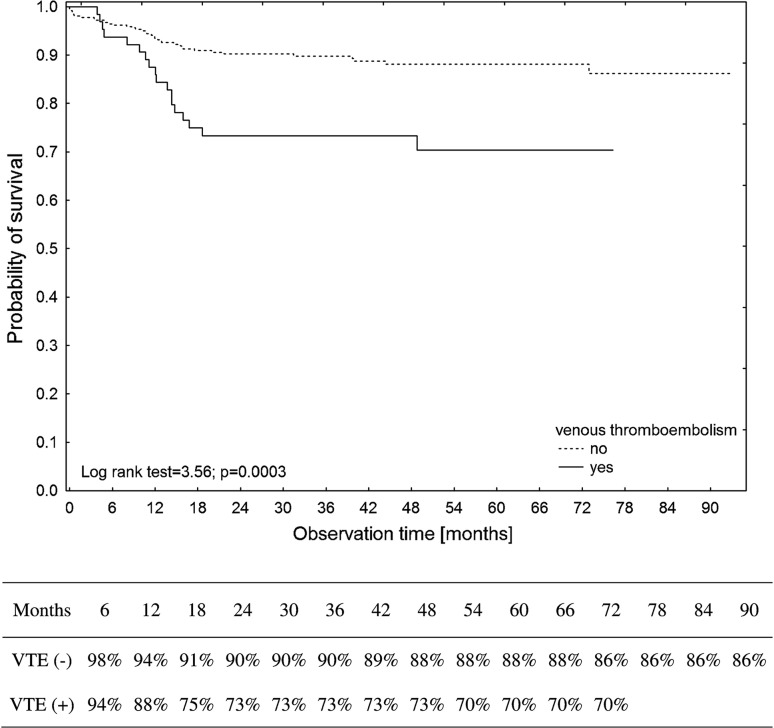
Kaplan–Meier estimates of the cumulative survival probability of the studied patients with or without venous thromboembolism

Patients who developed VTE had, according to the Kaplan–Meier method, significantly lower overall survival rates than patients without VTE (log-rank test = 3.56, *P* = 0.0003). Estimated 6-year survival rates of 86 and 70% were calculated for patients without and with VTE occurrence (Fig. 2). HL patients had significantly better VTE-free survival rates than DLBCL patients (log-rank test = 2.51, *P* = 0.0122). Patients with bulky disease had, according to the Kaplan–Meier method, significantly lower VTE-free survival rates than those without bulky disease (log-rank test = 4.50, *P* < 0.0001).

At the cutoff point for the high-risk category (score ≥ 3), we calculated the sensitivity (probability of high risk in those patients experiencing VTE), specificity (probability of high risk in those patients not experiencing VTE), positive predictive value (PPV, probability of high risk in those patients identified to be at high risk) and negative predictive value (NPV, probability of no VTE in those patients identified to be at low risk) for VTE development. For HL, the sensitivity was 37%, specificity was 81%, PPV was 19%, and NPV was 63%. For DLBCL, the sensitivity was 9%, specificity was 9%, PPV was 11%, and NPV was 91% (Table 3). For all subjects, the sensitivity was 17%, specificity was 85%, PPV was 15%, and NPV was 82% (*C* statistic 0.51). In patients treated for DLBCL and HL, the KRS model failed to be prognostic for VTE.

**Table 3:** VTE rates and negative and positive predictive values for the development of VTE based on the Khorana Risk Score in lymphoma patients

Risk group	Patients (*n*)	VTE (*n*)	PPV (%)	NPV (%)	Sensitivity (%)	Specificity (%)	*C* statistic
HL							
Intermediate	148	12	100	0	100	0	0.59
High	39	7	19	63	37	81	
DLBCL							
Intermediate	216	41	100	0	100	0	0.49
High	25	4	11	91	9	89	
Overall population							
Intermediate	364	53	100	0	100	0	0.51
High	64	11	15	82	17	85	

*PPV* positive predictive value, *NPP* negative predictive value, *VTE* venous thromboembolism

### Factors associated with VTE and overall survival

The DLBCL histological subtype of lymphoma, poor prognostic disease, the presence of mediastinal bulky disease and pre-chemotherapy leukocyte count > 11 × 10^9^/l were significantly associated with an increased risk of VTE based on the univariate analysis (Table 4). There was a trend toward an increased risk of VTE in patients with advanced disease and a pre-chemotherapy hemoglobin value < 10 g/dl. When variables were included in the multivariate analysis, DLBCL histological subtype (OR 1.61; 95% CI 1.17–2.19, *P* = 0.003), bulky disease (OR 2.34; 95% CI 1.62–3.36, *P* < 0.0001) and poor prognostic disease (OR 1.32; 95% CI 1.01–1.74, *P* = 0.049) remained significant for VTE development.

**Table 4:** Univariate analyses of determining factors that affect VTE development in patients with lymphoid malignancies

Factor	Univariate analysis
	Odds ratio (95% CI), *P*
Male gender	0.65 (0.38–1.12), 0.1208
Age	1.00 (0.98–1.11), 0.9610
Type of lymphoma: DLBCL	2.03 (1.14–3.61), 0.0157
Advanced disease^a^	1.61 (0.94–2.77), 0.0844
Poor prognostic disease^b^	0.54 (0.31–0.92), 0.0224
Constitutional symptoms	0.90 (0.52–1.55), 0.6940
Bulky disease	4.34 (2.21–8.53), 0.0001
High KRS^c^	1.06 (0.51–2.21), 0.8770
Pre-chemotherapy platelet count > 350 × 10^9^/l	1.15 (0.63–2.12), 0.6495
Pre-chemotherapy leukocyte count > 11 × 10^9^/l	1.81 (1.01–3.26), 0.0474
Pre-chemotherapy hemoglobin < 10 g/dl × 10^9^/l	0.48 (0.23–1.01), 0.0526

*CI* confidence interval, *IPI* International Prognostic Index

*P* < 0.05: statistically significant

^a^Advanced disease: stage according to Lugano IV

^b^IPI, International Prognostic Index ≥ 3; IPS, International Prognostic Score ≥ 3

^c^According to the Khorana Risk Score (KRS) for VTE risk assessment

During a median follow-up of 37 months, 56 patients (13%) died. In a Kaplan–Meier analysis of the probability of survival, HL patients had significantly higher overall survival rates than DLBCL patients (log-rank test = 2.66, *P* = 0.0079). No impact of a high KRS on prognosis was found (log-rank test = 1.18, *P* = 0.2387; Fig. 1b). No difference in overall survival rates was found between the patients with or without bulky disease (log-rank test = 0.79, *P* = 0.4281). In the Cox regression model, age, male gender, poor prognostic disease and the presence of VTE were significantly correlated with mortality (Table 5). Only a trend toward an association between high KRS and mortality was revealed.

**Table 5:** Factors that affected mortality in patients with DLBCL and HL according to the Cox proportional hazards model

Factor	Hazard ratio (95% CI)	*P* value
Age	1.03 (95% CI 1.01–1.05)	0.0011
Male gender	3.41 (95% CI 1.82–6.37)	0.0001
Type of lymphoma: DLBCL	1.35 (95% CI 0.65–2.77)	0.4181
Advanced disease^a^	1.52 (95% CI 0.79–2.91)	0.2089
Poor prognostic disease^b^	2.25 (95% CI 1.19–4.27)	0.0130
Bulky disease	0.77 (95% CI 0.25–2.35)	0.6509
High KRS^c^	2.05 (95% CI 0.99–4.23)	0.0501
Presence of VTE	2.15 (95% CI 1.19–3.86)	0.0106

*CI* confidence interval, *HR* hazard ratio, *IPI* International Prognostic Index

*P* < 0.05: statistically significant

^a^Advanced disease: stage according to Lugano IV

^b^IPI, International Prognostic Index ≥ 3; IPS, International Prognostic Score ≥ 3

^c^According to the Khorana Risk Score (KRS) for VTE risk assessment

## Discussion

This study provides additional evidence that the universal, non-specific VTE assessment model known as the KRS is not suitable for all types of malignancies, particularly lymphoid malignancies. Both DLBCL and HL require prompt initial systemic chemotherapy, and, as they are both lymphomas, they are categorized as intermediate groups according to the KRS. Consistent with the literature, our data show that the overall incidence rate of VTE in patients with lymphoid malignancies is high, reaching 15% within the observation time [15–18]. In addition, we demonstrated a difference in the VTE incidence between DLBCL and HL patients (19 vs. 10%) and in the clinical presentation.

The KRS was developed for the stratification of outpatients with heterogeneous cancer undergoing chemotherapy during a median observation period of 2.5 months according to their VTE risk [5]. It should be emphasized that while the KRS was developed based on cancer outpatients with different types of malignancies, several cancers are strongly associated with VTE, including brain tumors, and patients with a poor performance status were underrepresented [19, 20]. All patients in our study had an ECOG/WHO score of 0–2 and were eligible for outpatient chemotherapy regimens. First, the KRS model was validated in several prospective studies, including studies by the Vienna CATS group [6, 21] and a retrospective study with cisplatin-based chemotherapy in patients with different cancers [22]. In the present study, the KRS was not able to differentiate the risk of VTE events in patients with DLBCL and HL receiving outpatient chemotherapy. The KRS had a low positive prognostic value for the entire cohort (15%), as well as for HL (19%) and DLBCL (11%). For all subjects, the *C* statistic was lower than that in the Khorana validation study. Our results are in accordance with a Korean study of a prospective cohort of patients with DLBCL, in which the KRS failed to show an association with VTE [23]. However, our results are in contrast to those of the study by Santi et al. [24], which indicated that the KRS was predictive of VTE in a cohort of patients with various lymphomas. Furthermore, in a multicenter cohort of cancer patients, the KRS was not able to discriminate between patients with and without VTE; however, the addition of other biomarkers increased its predictive value [25]. Similar to the SAVE-ONCO and FRAGMATIC studies, in our study, VTE events occurred more often in the intermediate-risk KRS group than in the high-risk KRS group [26–29].

In our study of lymphoid malignancies, patients with bulky disease had a nearly 2.5-fold increased risk of VTE development based on the multivariate analysis. This parameter may be a specific risk factor for VTE development in patients receiving outpatient chemotherapy due to HL or DLBCL. Furthermore, an association between poor prognostic disease and VTE risk was found. This finding is in accordance with the study by Lim et al. [23]. In both our study and the literature, the incidence of VTE in DLBCL patients was higher than that in the HL group [15, 17, 18, 30–32]. In the multivariate analysis, patients with DLBCL-type lymphoma had a nearly 1.6-fold increased risk of VTE events compared with the HL group, demonstrating the differences in VTE risk within the lymphoma histological subtypes. Of the parameters in the KRS, only a pre-chemotherapy leukocyte count > 11x10^9^/l was significantly associated with VTE in the univariate analysis. Because the majority of our patients were diagnosed with an advanced stage of disease with constitutional symptoms that included weight loss, the value of a BMI parameter > 35 kg/m^2^ had limited application. Additionally, in an advanced stage, bone marrow involvement may lead to cytopenia, and the utility of the increased leukocyte count is reduced. None of our patients received erythropoietin-stimulating agents, which makes this KRS factor irrelevant. Other parameters also failed to show associations. Recently, some studies performed in different types of malignancies that are strongly associated with an increased risk of VTE, such as lung cancer and pancreatic cancer, failed to show the utility of the KRS [33, 34].

Our findings show the need for the identification of lymphoma-specific biomarkers, such as the presence of bulky disease, poor prognostic score and histological subtype of lymphoma, to adequately stratify or predict VTE events in patients with lymphoid malignancies. The closest biomarker is the ThroLy score developed by Antic et al. [35], which is not widely used and has not yet been independently validated. Although no prophylaxis is recommended in any guidelines, the risk of developing VTE in lymphoma patients undergoing chemotherapy is high, and it remains unclear whether a subgroup of high-risk patients could benefit from primary VTE prophylaxis [36, 37].

Previous studies have shown that cancer-associated thrombosis is a leading cause of death among patients with cancer, particularly those receiving outpatient chemotherapy [38]. Although the KRS was not developed to evaluate the risks of mortality, some studies indicate that the KRS may be associated with mortality in cancer patients [33, 39, 40]. First, Kuderer et al. [39] reported that the KRS is predictive of early mortality and cancer progression, independent of other prognostic factors, including VTE. The impact of a high KRS on early mortality in pancreatic adenocarcinoma was reported [40]. The preliminary data showed that KRS was a predictor of mortality in patients with lung cancer; however, in this cohort, a high KRS was not predictive of VTE [33]. Contrary to previous research, in the present study, the KRS was not able to predict mortality in patients with lymphoid malignancies. Consistent with the literature, our study shows that the patients who developed VTE had an overall twofold increased risk of mortality compared with patients in the non-VTE group [17, 41–44]. In contrast, Lim et al. [23] reported no difference in the overall survival rate between patients with or without VTE treated for DLBCL. Our results showed that advanced age, male gender and poor prognostic disease were prognostic for inferior survival in patients with lymphoid malignancies. These results are consistent with previous studies [15, 17, 45]. In our study, we were able to demonstrate the negative impact of VTE on all-cause mortality and to discriminate specific risk factors, such as the presence of bulky disease, for VTE development among lymphoma patients irrespective of the KRS.

The strengths of the present study include the following: (1) the recruitment of two groups of lymphoma patients with different histological subtypes, with both groups managed using the same diagnostic and treatment procedure in one hospital; (2) the size of the population; and (3) the long-term follow-up. Moreover, the differences in VTE risk and factors associated with VTE development between the HL and DLBCL groups were analyzed. The main limitation of the study is the retrospective collection of the data. There was no routine screening for VTE, and only symptomatic events were evaluated.

In conclusion, in this cohort of patients with DLBCL and HL, the KRS did not adequately stratify or predict VTE events in our patients at higher risk of VTE. In the multivariate analysis, DLBCL, bulky disease and poor prognostic disease were all significantly associated with VTE development. Our findings demonstrate the need for the identification of lymphoma-specific biomarkers and VTE risk assessment models. Furthermore, we found no association of the KRS with all-cause mortality. Additional prospective, large studies are needed to confirm or refute our findings before changes are implemented in clinical practice.
